# Antimicrobial Activity and Possible Mechanism of Action of Citral against *Cronobacter sakazakii*

**DOI:** 10.1371/journal.pone.0159006

**Published:** 2016-07-14

**Authors:** Chao Shi, Kaikuo Song, Xiaorong Zhang, Yi Sun, Yue Sui, Yifei Chen, Zhenyu Jia, Huihui Sun, Zheng Sun, Xiaodong Xia

**Affiliations:** 1 College of Food Science and Engineering, Northwest A&F University, Yangling, Shaanxi, China; 2 Sino-US Joint Research Center for Food Safety, Yangling, Shaanxi, China; University of Padova, Medical School, ITALY

## Abstract

Citral is a flavor component that is commonly used in food, beverage and fragrance industries. *Cronobacter sakazakii* is a food-borne pathogen associated with severe illness and high mortality in neonates and infants. The objective of the present study was to evaluate antimicrobial effect of citral against *C*. *sakazakii* strains. The minimum inhibitory concentration (MIC) of citral against *C*. *sakazakii* was determined via agar dilution method, then Gompertz models were used to quantitate the effect of citral on microbial growth kinetics. Changes in intracellular pH (pH_in_), membrane potential, intracellular ATP concentration, and membrane integrity were measured to elucidate the possible antimicrobial mechanism. Cell morphology changes were also examined using a field emission scanning electron microscope. The MICs of citral against *C*. *sakazakii* strains ranged from 0.27 to 0.54 mg/mL, and citral resulted in a longer lag phase and lower growth rate of *C*. *sakazakii* compared to the control. Citral affected the cell membrane of *C*. *sakazakii*, as evidenced by decreased intracellular ATP concentration, reduced pH_in_, and cell membrane hyperpolarization. Scanning electron microscopy analysis further confirmed that *C*. *sakazakii* cell membranes were damaged by citral. These findings suggest that citral exhibits antimicrobial effect against *C*. *sakazakii* strains and could be potentially used to control *C*. *sakazakii* in foods. However, how it works in food systems where many other components may interfere with its efficacy should be tested in future research before its real application.

## Introduction

*Cronobacter sakazakii* is a foodborne pathogen [1] that has been implicated in some severe forms of neonatal infection including bacteraemia, necrotizing enterocolitis (NEC), and infant meningitis [2]. The infant mortality rate associated with *C*. *sakazakii* infections is quite dangerously high, ranging from 50–80% [3]. In addition to those infections in neonates, diseases caused by *C*. *sakazakii* infection have been reported for almost every age group [4]. The International Commission for Microbiological Specifications for Foods (2002) ranked *C*. *sakazakii* as a “severe hazard for restricted populations, life threatening or substantial chronic sequelae of long duration” [5].

Foodborne diseases have increasingly become a worldwide public health concern. In addition to different food processing for reducing or eliminating spoilage or pathogenic microorganisms in food, synthetic preservatives are extensively used in food industry to increase shelf-life or enhance food safety [6]. But there is currently enthusiasm for search of natural antimicrobials for use in food industry due to many reasons. For instance, imprudent use of antibiotics in husbandry has led to increasing resistance in foodborne pathogens [7]; And consumers prefer to foods with less synthetic preservatives for fear of their potential negative health effects [6]. All of these have stimulate research on natural antimicrobials with an intention to reduce the usage of antibiotic and synthetic preservatives. Plant-derived compounds have been considered to be important alternative, consumer accepted and widely available sources of natural antimicrobials [8].

Citral (C_10_H_16_O) is one of the most common flavor compounds found in citrus oils, which has been already widely used in foods and beverages (e.g., soft drinks and desserts) [9]. Citral is a monoterpenoid aldehyde [10] often present in the form of stereoisomers geranial and neral [11] that has been identified in the leaves and fruits of several plant species including myrtle trees, basil, lemon, lime, lemongrass, orange, and bergamot [10,12]. A number of experimental and clinical studies have shown that citral has favorable anti-inflammatory [13] and anti-corrosive [14] effects, and there is increasing evidence that citral acts as a fungicidal and bactericidal agent [15].

Although citral has been reported to be effective against a variety of microbial species, there have been no reports on its antimicrobial activity against *C*. *sakazakii* and possible mode of action. To fill this gap, the aim of the present study was to determine antimicrobial effect of citral against *C*. *sakazakii*. We also investigated possible antimicrobial mechanisms by measuring changes in intracellular ATP concentrations, membrane potential, intracellular pH (pH_in_), and membrane integrity, as well as changes in cell membrane microstructures.

## Materials and Methods

### Reagents

Citral (CAS:5392-40-5) was obtained from Chengdu Must Bio-technology Co., Ltd (Chengdu, Sichuan, China) at a HPLC purity of at least 99.2%. Sample solution was prepared in ultrapure sterilized water and sterilized by filtration immediately before use to minimize any oxidation of the compound. All other chemicals were of analytical grade and used as-received.

### Bacterial strains and culture conditions

*C*. *sakazakii* strains ATCC 29544 and ATCC 29004 were purchased from the American Type Culture Collection (ATCC, Manassas, VA, USA). Three other *C*. *sakazakii* strains (CS 1, CS 2, CS 3) were taken from our laboratory strain collection, which were originally isolated from infant formula and infant rice cereal collected from supermarkets in China. All isolates were used in minimum inhibitory concentration assay and only *C*. *sakazakii* ATCC 29544 was used for antimicrobial mechanism analysis. All strains were stored in tryptone soya broth (TSB) with 20% glycerol (v/v) at -80°C. Before each experiment, stock cultures were streaked on tryptone soya agar (TSA) and grew at 37°C for 18 h. Then a loopful of each strain was inoculated into 30 mL TSB and incubated for 18 h at 37°C.

### Minimum inhibitory concentration (MIC) determinations

The MIC of citral was determined by agar dilution method as described by the European Committee for Antimicrobial Susceptibility Testing [16] with some modifications. *Escherichia coli* ATCC 25922 was used as a quality control. While determining the antimicrobial activity of citral, ampicillin was used as a positive reference standard for all test strains. The stock antibiotic solutions were prepared in sterile water, then sterilized through a 0.22 μm Acrodisc filter (Gelman, USA). Then TSA was aseptically transferred into sterile 24-well plates containing either citral or the antibiotic. The content (final volume 500 μL) of each well was gently mixed. The final concentrations of citral were 0, 0.07, 0.135, 0.27, 0.41, 0.54, 0.81, and 1.08 mg/mL, and that of ampicillin was 0.1 mg/mL. After hardening, the TSA was spotted with 2 μL (approximately 10^4^ CFU) of the tested bacterium. The spots were left to dry, then the plates were incubated at 37°C for 24 h. The lowest concentration of citral at which no visible growth of test organisms occurs was defined as the MIC.

### Growth curves and kinetic parameters

The growth curves in TSB at 37°C were determined as previously described [17]. *C*. *sakazakii* strain ATCC 29544 was grown to an OD_600_ value of 0.1 in TSB, then 125 μL of the culture was transferred into each well of a 96-well microtiter plate (Nunc, Copenhagen, Denmark). Citral was added to the cultures to obtain final concentrations of 1/16MIC, 1/8MIC, 1/4MIC, 1/2MIC, and MIC, and TSB was used as a negative control. Bacteria were further cultured at 37°C, and cell growth was monitored at 600 nm using a multimode plate reader (Tecan, Infinite^™^ M200 PRO, Männedorf, Switzerland).

The model used to fit growth curves to the data as-obtained was comprised of a modified Gompertz equation as follows:

$${\text{OD}}_{\text{t}}=A\text{+(}B-A\text{)exp}\left\{-\text{exp}\left[-\mu \left(t-M\right)\right]\right\}$$

$$\lambda =M-\left(\text{1}/\mu \right),\text{ and }{\text{u}}_{\text{max}}=\left(B-A\right)\mu /\text{e}$$

where OD_t_ is optical density at 600 nm (OD_600_) at time *t*, *t* is the time (in hours) that had elapsed since incubation, *B* is the maximum OD_600_, *A* is the initial OD_600_, *M* is the time (in hours) of the inflexion point in the exponential phase of model function, μ is the relative growth rate at time *M*, λis the lag time (i.e., the time until the lag period ended,) and μ_max_ is the maximum growth rate achieved (ΔOD_600_ per hour.) The model was evaluated for goodness of fit according to the coefficient of determination *R*^2^.

### Intracellular ATP concentrations

The method described by Sanchez et al. [18] was followed (with slight modification) to determine intracellular ATP concentration. Briefly, the overnight culture of *C*. *sakazakii* strain ATCC 29544 was centrifuged for 5 min at 5000 × g and the supernatant was removed, the cell pellets were washed three times with 0.1 mol/L of phosphate buffered saline (PBS, pH 7.0), then the cells were collected by centrifugation under identical conditions. A cell suspension (OD_600_ = 0.5) made with 50 mL of PBS and 2 mL of the cell suspension was placed into a 2.5 mL Eppendorf tube for citral treatment. Citral was added to each tube to achieve final concentrations of 0 (control), MIC, and 2MIC, respectively. Then the samples were maintained at 37°C for 30 min. To extract the ATP, cells were lysed on ice by ultrasound, and centrifuged at 5000 × g for 5 min. The top layer was retrieved and stored on ice to prevent ATP loss until measurement. Intracellular ATP was measured with an ATP assay kit (Beyotime Bioengineering Institute, Shanghai, China). After adding 125 μL of ATP assay mix to 125 μL of supernatant in white, opaque, 96-well microtiter plates, the supernatant ATP concentrations were measured with a microplate reader (Tecan, Infinite^™^ M200 PRO, Männedorf, Switzerland) and recorded as the intracellular ATP concentration.

### Intracellular pH_in_ measurements

Intracellular pH was determined according to Breeuwer et al. with slight modification [19]. To load a fluorescent probe in the sample cells, 250 μL of overnight cultures of *C*. *sakazakii* strain ATCC 29544 was transferred into TSB (30 mL) and incubated at 37°C for 8 h (approximately OD = 0.6 at 600 nm). Cells were then harvested by centrifugation (5000 × g, 10 min) and washed twice with 50 mM HEPES buffer (containing 5 mM EDTA, pH 8.0). Then cell pellets were re-suspended in 20 mL of the same buffer. Next, 3.0 μM of the probe, carboxyfluorescein diacetate succinimidyl ester (cFDA-SE; Molecular Probes, Sigma, Louis, USA), was added. Then cells were incubated for 20 min at 37°C, washed once in 50 mM potassium phosphate buffer with 10 mM MgCl_2_ (pH 7.0), and re-suspended in 10 mL of buffer. To eliminate non-conjugated cFDA-SE, glucose (10 mM, final concentration) was added and the cells were incubated for an additional 30 min at 37°C. Finally, cells were washed twice, re-suspended in 50 mM PBS (pH 7.0), and stored on ice.

An aliquot of cell suspension labeled by fluorescence was dispensed into tubes with citral at three different concentrations (0, MIC, 2MIC), then transferred into black, opaque, 96-well microtiter plates. After treatment for 20 min, fluorescence intensities were measured under two excitation wavelengths, 440 nm and 490 nm, while the monochromator was rapidly alternated between the wavelengths. The emission was collected at 520 nm, where excitation and emission slit widths were 9 and 20 nm, respectively. The pH_in_ of the bacteria was determined according to the ratio of the fluorescence signal at the pH-sensitive wavelength (490 nm) and the fluorescence signal at the pH-insensitive wavelength (440 nm). All measurements were carried out on a microplate reader (Tecan, Infinite^™^ M200 PRO, Männedorf, Switzerland). During the assay, the system was maintained at 25°C. The fluorescence of the cell-free controls was deducted from the values of the treated samples.

Calibration curves were determined for cFDA-SE loaded cells with buffers of different pH. Buffers consisted of glycine (50 mM), citric acid (50 mM), Na_2_HPO_4_•2H_2_O (50 mM), and KCl (50 mM). pH of buffers were adjusted with either NaOH or HCl to various values (3, 4, 5, 6, 7, 8, 9, and 10). After equilibration of the pH_in_ and pH_out_ by adding valinomycin (10 μM) and nigericin (10 μM), fluorescence intensity was measured at 25°C.

### Membrane potentials

The methods described by Sanchez et al. was used with slight modification to examine membrane potentials [18]. Briefly, cells were grown in 30 mL of TSB at 37°C to an optical density of 0.5 at 600 nm, then harvested by centrifugation (5000 × g, 5 min) and washed twice with PBS (pH 7.0). Next, 125 μL of cell suspensions were placed in black, opaque, 96-well microtiter plates for 30 min at 37°C, then 1 μM of the membrane potential-sensitive fluorescent probe bis-(1, 3-dibutylbarbituric acid) trimethine oxonol (DiBAC_4_ (3); Molecular Probes, Sigma, Louis, USA) was added and incubated for 30 min at 37°C, followed by addition of citral at three concentrations (0, MIC, 2MIC). Fluorescence was then measured on a fluorescence microplate reader (Tecan, Infinite^™^ M200 PRO, Männedorf, Switzerland) at excitation and emission wavelengths 492 and 515 nm, respectively. The excitation and emission slit widths were 3 and 5 nm, respectively. Background fluorescence resulting from the medium was determined and the results corrected as necessary.

### Bacterial membrane integrity

Cell membrane integrity was determined according to the method described by Alakomi et al. with slight modification [20], using the LIVE/DEAD^®^ *Bac*Light^™^ Bacterial Viability Kit (Molecular Probes, Eugene, OR). The overnight culture of *C*. *sakazakii* strain ATCC 29544 was harvested by centrifugation (10,000 × g, 15 min), then the supernatant was removed and pellets re-suspended in 2 mL of 0.85% NaCl. To acquire viable and non-viable cells, 1 mL of the suspension was added to each of two 50 mL centrifuge tubes containing either 20 mL of 0.85% NaCl (live bacteria) or 20 mL of 70% isopropyl alcohol (dead bacteria). After incubating both samples at room temperature for 1 h (while mixing every 15 min), both samples were pelleted by centrifugation twice at 10,000 × g for 10 minutes, then optical density was adjusted at 600 nm to 0.5 and five viable cells’ proportions (0, 10%, 50%, 90%, and 100%) were mixed to obtain standard samples. A 2X working stain solution was prepared by mixing equal volumes of SYTO 9 and propidium iodide (PI) and adding the mixture into 2 mL of filter-sterilized dH_2_O.

Cells treated with citral (0, MIC, 2MIC) at 37°C for 15 min were quickly centrifuged (11,000 × g, 1 min), then 100 μL of samples were pipetted in three respective parallel samples into black, opaque, 96-well microtiter plates. Aliquots of 100 μL of the 2X staining solution were then added to each well and mixed completely. The plate was then incubated at 25°C in the dark for 15 min before measurement of the fluorescence of bacterial suspensions with a fluorescence microplate reader (Tecan, Infinite^™^ M200 PRO, Männedorf, Switzerland). The excitation/emission maxima for the dyes were 485/542 nm for SYTO 9 and 485/610 nm for PI. Cell suspension without citral treatment served as the control.

### Field emission scanning electron microscopy (FESEM)

The FESEM assay was carried out accordingly to a previously published method with slight modification [21]. Cells (OD_600_ = 0.5) were treated with citral at 0, MIC, and 2MIC, then after being incubated at 37°C for 2 h, the cells were harvested by centrifugation for 10 min at 5000 × g, washed twice with PBS (pH 7.0), and re-suspended in water containing 2.5% glutaraldeyde then kept at 4°C for 12 h to fix the cells. After centrifugation, the cells were dehydrated in water-alcohol solutions with different concentrations of alcohol (30%, 50%, 70%, 80%, 90%, 100%) for 10 min each. The samples were finally fixed on FESEM supports, sputter-coated with gold under vacuum conditions, and examined under a scanning electron microscope (S-4800, Hitachi, Tokyo, Japan).

### Statistical analysis

All experiments were performed in triplicate. Statistical analyses were performed in SPSS software (Version 19.0; SPSS, Inc., Chicago, IL). The data are presented as the mean ± SD (n = 3). Differences between means were tested by Student’s t-test. Differences were defined as significant at *P* ≤ 0.05.

## Results

### MIC of citral against *C*. *sakazakii* strains

Citral showed inhibitory effects against all five *C*. *sakazakii* strains tested (Table 1). The MICs of citral against ATCC 29544, ATCC 29004 and CS 1 were 0.54 mg/mL, and the MICs against isolates CS 2 and CS 3 were 0.27 mg/mL. And only *C*. *sakazakii* ATCC 29544 was selected for further study.

**Table 1:** Minimum inhibitory concentrations of citral against different strains of *C*. *sakazakii*.

Strain	Origin	MIC (mg/mL)
ATCC 29544	Child's throat	0.54
ATCC 29004	Infant formula	0.54
CS1	Infant formula	0.54
CS2	Infant rice cereal	0.27
CS3	Infant rice cereal	0.27

### Effect of citral on *C*. *sakazakii* growth

**Fig 1:**
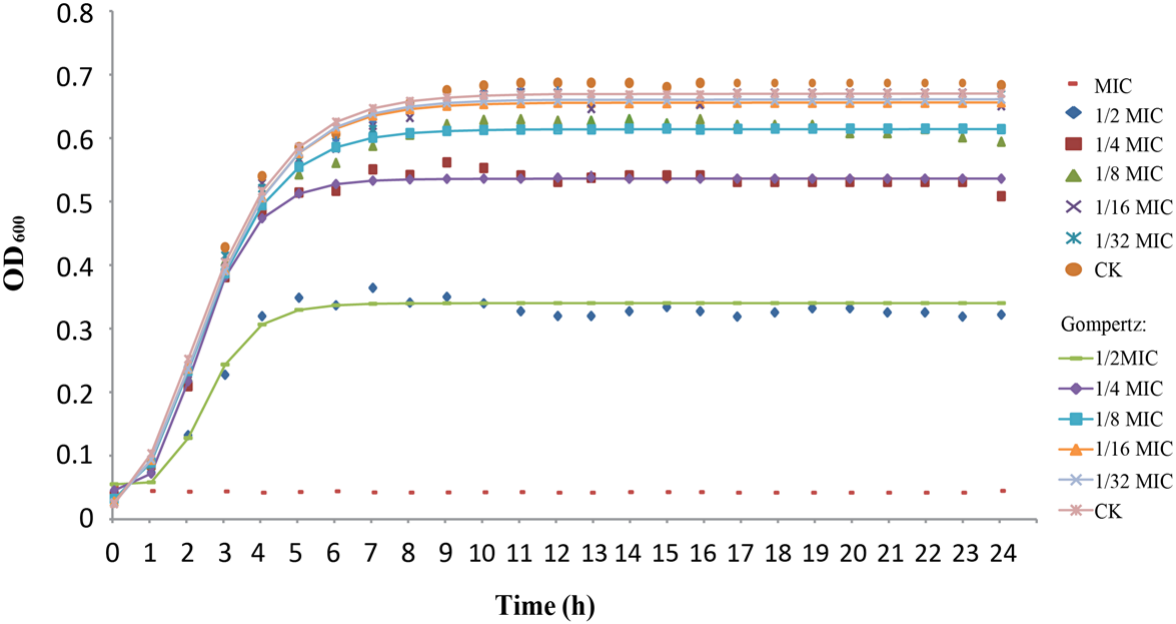
Growth curves of *C*. *sakazakii* ATCC 29544 cultured in TSB with various concentrations of citral. The lines represent the fit of the experimental data to the modified Gompertz model. Bars represent the standard deviation (n = 3).

*C*. *sakazakii* growth in TSB was fitted to the modified Gompertz equation (Fig 1), where high R^2^ values (> 0.996) indicate good fitness of the model. By comparison of different growth curves, it was demonstrated that higher concentrations of citral resulted in longer lag phase and lower growth rate compared to lower citral concentrations (Table 2).

**Table 2:** Kinetic parameters of *C*. *sakazakii* cells grown in TSB with different concentration of citral.

Concentration of citral	λ±SE	μ_max_±SE	OD_max_
1/2 MIC	1.424±0.006^A^	0.125±0.001^A^	0.340±0.001^A^
1/4 MIC	0.814±0.002^B^	0.147±0.002^B^	0.536±0.002^B^
1/8 MIC	0.745±0.003^C^	0.164±0.003^C^	0.614±0.001^C^
1/16 MIC	0.660±0.002^D^	0.163±0.003^C^	0.656±0.001^D^
1/32 MIC	0.631±0.002^E^	0.161±0.003^C^	0.661±0.001^E^
0 (CK)	0.498±0.001^F^	0.162±0.001^C^	0.670±0.001^F^

λ, lag phase (in hours); μ_max_, maximum growth rate (in OD per hour); OD_max_, maximum optical density (determined at 600 nm).

Mean values in the same column followed by different letters are statistically different (*P* ≤ 0.05) (n = 6).

### Effect of citral on intracellular ATP concentrations

**Fig 2:**
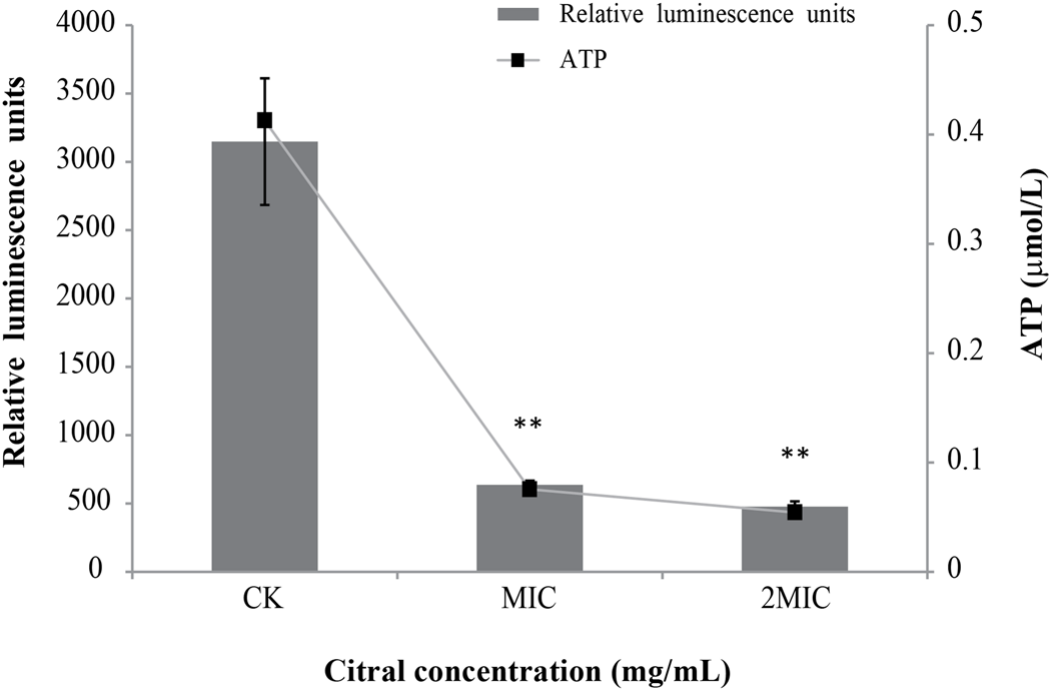
Effects of citral on intracellular ATP production by *C*. *sakazakii* ATCC 29544. Values represent the means of triplicate measurements. Bars represent the standard deviation (n = 3). **P ≤ 0.01.

There was a good linearity between relative luminescence units and ATP concentration (y = 7445.5x + 74.167; R^2^ = 0.999). The level of intracellular ATP of *C*. *sakazakii* decreased significantly (*P* ≤ 0.01) as citral concentration increased (Fig 2). The original ATP concentration of *C*. *sakazakii* ATCC 29544 was 0.41 μmol/L. Addition of citral at MIC and 2MIC caused ATP concentrations reduced to 0.075 and 0.054 μmol/L respectively.

### Effect of citral on pH_in_

**Fig 3:**
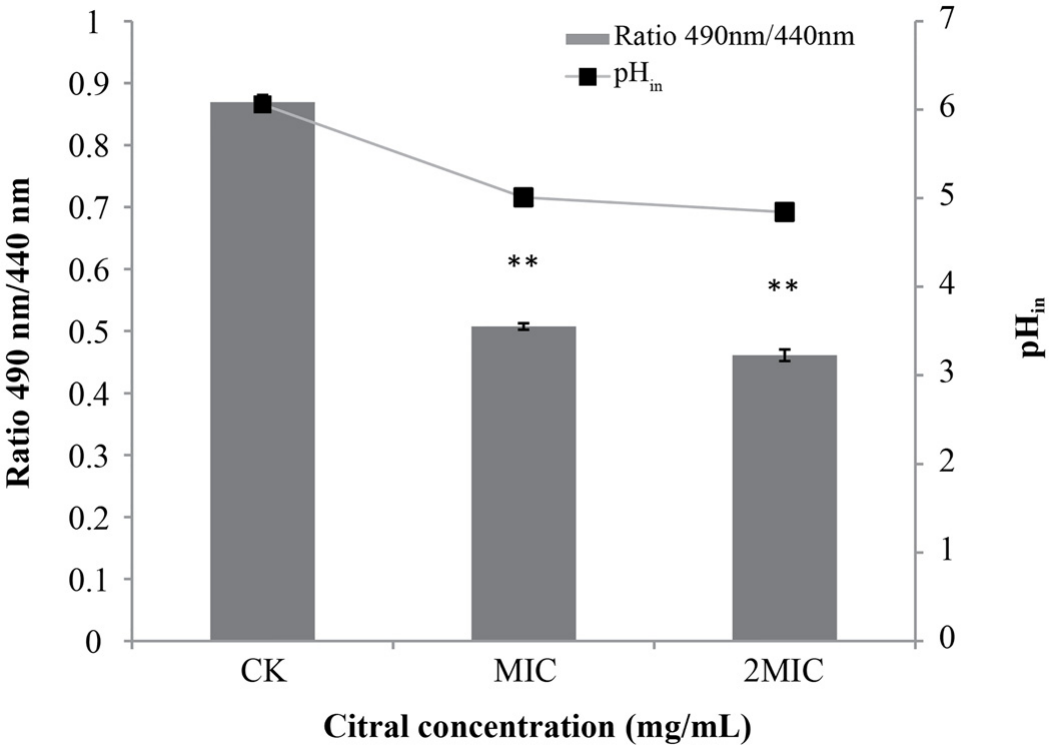
Effects of citral on the intracellular pH of *C*. *sakazakii* ATCC 29544. Values represent the means of triplicate measurements. Bars represent the standard deviation (n = 3*)*. **P ≤ 0.01

A clear change in intracellular pH was found after citral addition (Fig 3). The pH_in_ of *C*. *sakazakii* ATCC 29544 was 6.06 ± 0.03. The addition of citral at MIC caused a significant (*P* ≤ 0.01) decrease in pH_in_ of *C*. *sakazakii*, from 6.06 ± 0.03 to 5.01 ± 0.02. The addition of citral at 2MIC further decreased (*P* ≤ 0.01 compared to control) the pH_in_ to 4.84 ± 0.03.

### Effect of citral on membrane potential

**Fig 4:**
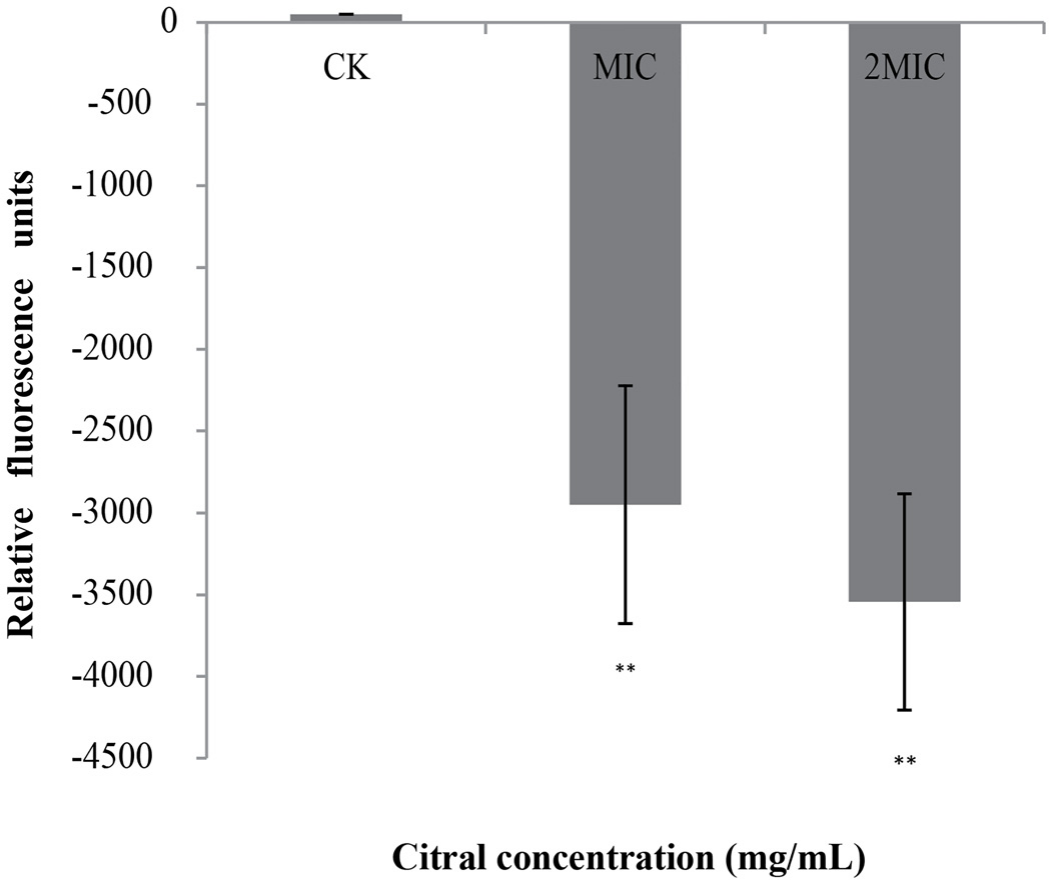
Effects of citral on the membrane potentials of *C*. *sakazakii* ATCC 29544. Negative values indicate hyperpolarization. Values represent the means of triplicate measurements. Bars represent the standard deviation (n = 3*)*. **P ≤ 0.01.

Cells treated with citral showed hyperpolarized cell membranes, as evidenced by decreases (i.e., negative values) in fluorescence (Fig 4). We observed more decreases in fluorescence as citral concentration increased from MIC to 2MIC.

### Fluorimetric detection of cell membrane injury

There was a good linearity between green fluorescent intensity and the percentage of viable bacteria (y = 26564x + 14473; R^2^ = 0.9931). Both tested concentrations of citral caused a significant reduction in viable cell fluorescence. Citral at MIC caused a 56% reduction in cell fluorescence, and citral at 2MIC caused a 85% reduction (Table 3).

**Table 3:** Reduction of viable cell fluorescence of *C*. *sakazakii* ATCC 29544 treated with citral at various concentrations.

Concentration of citral	Green fluorescence units	Percent viable cell fluorescence	Percent reduction of viable cell fluorescence
0 (CK)	40356	97%	3%
MIC	26299	44%	56%
2MIC	13381	15%	85%

### FESEM observations

**Fig 5:**
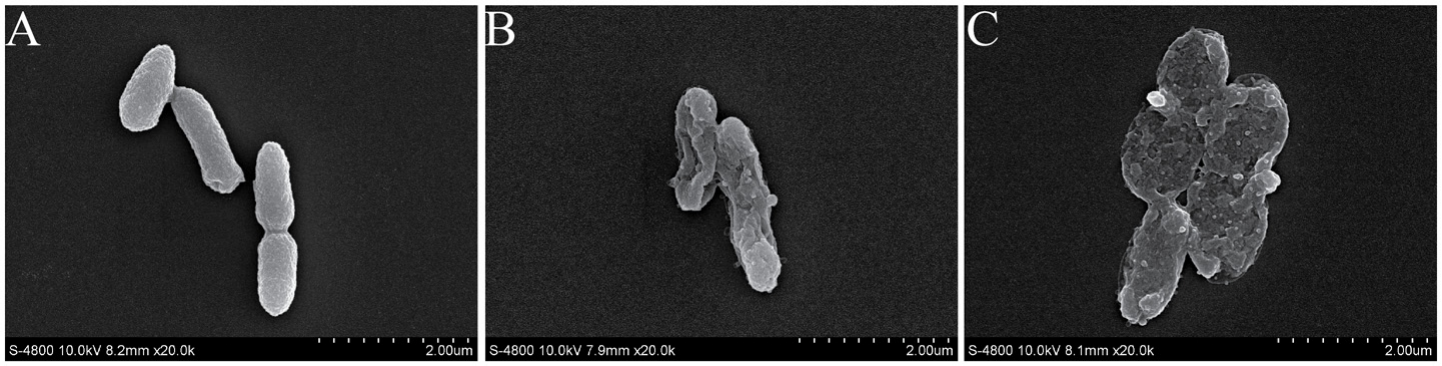
Scanning electron micrographs of *C*. *sakazakii* ATCC 29544 untreated (A), treated with citral at MIC for 2 h (B), and treated with citral at 2MIC for 2 h (C).

Cell morphology changes were examined by FESEM. Untreated *C*. *sakazakii* cells are shown in Fig 5A, and there were significant morphological differences between treated cells (Fig 5B and 5C) and the control. Untreated *C*. *sakazakii* cells exhibited a structure typical of gram-negative bacilli, with an undulating appearance, while the outer membrane of cells exposed to citral at MIC possessed a more wrinkled surface (Fig 5B). Those cells treated with citral at 2MIC showed substantial surface collapse of obvious cell wall damage due to disruption of cell wall permeability. The number of damaged cells and degree of damage increased as citral concentration increased.

## Discussion

Natural antimicrobials have gained more and more attention because of their effectiveness as well as growing demand for preservative-free food products [22]. Plant-derived essential oils including citral have been traditionally used to preserve foods and enhance food flavor [23]. Citral is currently a natural flavoring approved by the U.S. Food and Drug Administration (FDA) that is generally recognized as safe (GRAS 182.10). Citral has been reported to exhibit antimicrobial activity against pathogenic and food-spoilage bacteria such as *E*. *coli* O157:H7, *Salmonella* Typhimurium, *Listeria monocytogenes*, and *Staphylococcus aureus* [24,25]. However, its activity against *C*. *sakazakii* and how they exert their antibacterial activity have not yet been completely understood [17]. In this study, we investigated the effect of citral against *C*. *sakazakii* and determine citral’s mode of action by measuring changes in intracellular ATP concentration, membrane potential, intracellular pH (pH_in_), membrane integrity, and cell morphology.

Citral was shown be effective against five *C*. *sakazakii* strains either from clinical or food source, which may suggest its general effectiveness against *C*. *sakazakii* strains. Due to the limited number of strains tested in this study, more strains from clinical or environmental sources should be tested in the future study. Several reports have determined the antimicrobial effect of plant-derived compounds against *C*. *sakazakii* strains. The MICs of carvacrol and thymol were 0.19 mg/mL, and the MIC of cinnamic acid was higher than 0.8 mg/mL [26]. And it was previously shown that the MICs of lipoic acid against *C*. *sakazakii* strains ranged from 2.5 to 5.0 mg/mL [27]. In addition, tea polyphenols at 5 mg/mL could eliminate approximately 7.0 log CFU/mL of *C*. *sakazakii* within 1 h [28]. Comparing with most of these plant-derived compounds, citral exhibited a stronger antimicrobial activity against *C*. *sakazakii*.

ATP, one of the most important small molecules in living organisms, plays a crucial role in many cellular functions that are necessary for growth, replication, and survival. Intracellular ATP is necessary for storing and supplying metabolic energy, as well as for enzymatic reactions and signaling functions [29]. Citral significantly reduced the intracellular ATP of *C*. *sakazakii* (Fig 2). In a similar study, chlorogenic acid was shown to decrease the level of intracellular ATP in *Staphylococcus aureus* cells [21]. The reduction of intracellular ATP of *C*. *sakazakii* may be attributable to an increased rate of ATP hydrolysis inside the cells [18], or to increased membrane permeability which can cause intracellular ATP leakage through defective cell membranes [30].

Resting membrane potential is one of the most important parameters of living cells [31], as it relates closely to cell antibiotic uptake and bactericidal action [32]. We investigated the effect of citral on *C*. *sakazakii* membrane potential by applying DiBAC_4_ (3), which is an anionic fluorescent membrane potential dye that indicates changes in membrane polarization [33]. Upon cell depolarization, the dye enters the cells and fluorescence emitted by the dye is thus enhanced by bonding activation [34]. We found that *C*. *sakazakii* displayed rapid cell membrane hyperpolarization after exposure to citral. Sanchez et al. reported that basil, white sagebrush, and sweet acacia extracts caused a hyperpolarization of bacterial cellular membranes [18]. Studies have suggested that hyperpolarization occurs first due to change in pH, and second due to homeostasis of the cell membrane when K^+^ diffuses externally through K^+^ channels to balance the conductivity of superficial charges [31].

Many cellular processes are dependent on pH_in_, including DNA transcription, protein synthesis, and enzyme activities [35]. The cFDA-SE technique for measuring the pH_in_ of bacteria is based on identifying the intracellular conjugation of the cFSE succinimidyl group via the aliphatic amines of intracellular proteins, followed by the elimination of the free probe after a short incubation in the presence of glucose [36]. We found that citral induced changes in pH_in_ of *C*. *sakazakii* strains. Turgis et al. similarly demonstrated that the addition of mustard essential oil significantly lowers pH_in_ from 6.23 to 5.20 in *Escherichia coli* O157:H7 and from 6.59 to 5.44 in *Salmonella typhi* [37]. And reduced pH_in_ is reported be indicative of membrane damage [18].

Cell membrane structures are examined by FESEM and we found that citral caused severe morphological alterations in the cell membrane and cell wall. Previously such morphological alterations have been observed for various kinds of microbial pathogens. Di Pasqua et al. used SEM and found that eugenol is able to disrupt the *E*. *coli* O157:H7 membrane [38], allowing the leakage of intracellular constituents, while other compounds (carvacrol, cinnamaldehyde, limonene, and thymol) cause just structural alteration of the outer membrane. Rhayour et al. found that the effects of clove essential oils on membrane morphology vary among different strains [39]. In *E*. *coli* cells, holes are observed in the cell envelope, while in *Bacillus subtilis* cells, only cells with deformed shape is observed. In this study, *C*. *sakazakii* treated with citral exhibited large surface collapse without cell disintegration. This suggest that citral may bind to the cell surface and then penetrate to the target sites and interact with cell target molecules [30].

In summary, citral has antimicrobial activity against *C*. *sakazakii* and it exerts its effect by inducing changes in ATP concentration, cell membrane hyperpolarization, and reduction in cytoplasmic pH. These findings indicate the potential application of citral to control *C*. *sakazakii* in foods. More research on the interference of food components with citral and citral’s influence on organoleptic properties of food are among those issues that need to be resolved before its application in food systems, either alone or combined with other preservation technologies.
